# Using SDPC for Visual Exploratory Analysis of Semiconductor Production Line Sensor Data

**DOI:** 10.3390/s25071984

**Published:** 2025-03-22

**Authors:** Xinxiao Li, Xian-Hua Han, Yongqing Sun

**Affiliations:** 1 Faculty of Information Science, Shonan Institute of Technology, 1-1-25 Tsujido Nishikaigan, Fujisawa City 251-8511, Japan; 2 Graduate School of Artificial Intelligence and Science, Rikkyo University, 3-34-1 Nishi-Ikebukuro, Toshima-ku, Tokyo 171-8501, Japan; 3 College of Humanities and Sciences, Nihon University, 3-25-40 Sakurajosui, Setagaya-ku, Tokyo 156-8550, Japan; nakahara.eisei@nihon-u.ac.jp

**Keywords:** semiconductor production line, sensor data, visual exploratory analysis, parallel coordinates, superhigh-dimensional data

## Abstract

Vast amounts of data are continuously collected through sensors fitted into various pieces of equipment and processes in semiconductor production lines. These integrated datasets often encompass tens of thousands of dimensions, making it challenging to identify complex relationships among data dimensions for diagnosing defects and achieving high yield rates. Parallel Coordinate Plots (PCPs) are effective for visually analyzing multi-dimensional data, but traditional axis reordering methods struggle with superhigh-dimensional datasets. To address these challenges, we propose SDPC, an interactive PCP-based visual analysis system specifically tailored to the unique requirements of semiconductor production lines. SDPC employs a server–client architecture that efficiently visualizes sensor data in real time by dynamically selecting dimensions and down-sampling data based on user interactions. This enables engineers to explore high-dimensional sensor data without noticeable delays, enhancing their ability to identify defects quickly. By integrating user-defined filter conditions and focusing on defect-relevant dimensions, SDPC enhances interpretability and accelerates root cause identification. An evaluation with semiconductor production engineers demonstrated SPDC’s ability to facilitate real-time exploratory analysis, boost operational efficiency, reduce visual analysis time by two-thirds for on-site engineers, and ultimately lead to more effective production processes.

## 1. Introduction

Semiconductor production equipment, such as wafer manufacturing, lithography, and etching machines, is essential to the semiconductor manufacturing process. Each piece of equipment is equipped with numerous sensors, ensuring accuracy and efficiency at every stage of production. Each product passes through multiple pieces of equipment during the manufacturing process. Data collected from the sensors is invaluable for analyzing yield issues and identifying the root causes of defects in semiconductor manufacturing. These datasets include both numerical and categorical data but are not necessarily time-series data. Production line engineers can leverage this data to promptly implement corrective actions, reducing defect occurrence and enhancing overall production efficiency. The interplay between these sensors ensures the precise identification of anomalies and aids in defect detection. The vast amount of data from sensor networks, capturing equipment data, production data, quality data, environmental data, and process parameters spans tens to hundreds of thousands of dimensions.

The exponential growth of data space with increased dimensions leads to sparsity, making it difficult to find meaningful patterns and relationships in the data. High-dimensional data may include noisy, irrelevant, or redundant features. Identifying and removing these features is crucial but can be challenging, impacting the accuracy and reliability of the analysis. In addition, high-dimensional data analysis often results in complex models that are difficult to interpret.

There are various methods to analyze high-dimensional data. PCA [1] is used to reduce the dimensionality of the data and extract important features, making visualization and analysis easier. t-SNE [2] and UMAP [3] embed high-dimensional data into a low-dimensional space, helping to visualize the data structure. Techniques like K-means [4] and DBSCAN [5] are used to group data and discover patterns. However, these methods alter the original meaning of each dimension, reducing clarity and interpretability for users. As a result, they are not well-suited for our use case, which requires preserving the integrity and meaning of each dimension for effective analysis.

Combining visual exploration with domain knowledge is widely recognized as an efficient approach for mitigating potential drawbacks in data analysis. Unlike general-purpose visualization tools, manufacturing domain experts prefer direct access to raw sensor data to identify the root causes of defects. Their understanding of the manufacturing process enables them to interpret visualizations more effectively and extract meaningful insights.

Among various visualization techniques designed for sensor data analysis, parallel coordinates are particularly valued in semiconductor manufacturing environments. This technique enables users to leverage their expertise during the analysis process by representing high-dimensional data in two-dimensional spaces. Parallel coordinates facilitate the identification of patterns, correlations, and anomalies that might remain undetected with other visualization methods. This clarity and ease of interpretation are especially beneficial in complex manufacturing processes, where quick and accurate decision-making is essential.

The parallel coordinates technique has been widely used in multivariate analysis due to its uniform appearance and interaction of each dimension. Dama et al. [6] used parallel coordinates to visualize bioacoustics annotations in long-duration audio data. Maha and Orland [7] employed geo-coordinated parallel coordinates to disclose complex temporal and geospatial relations. Based on the parallel coordinates and making use of a tiled organization, Caat et al. [8] simultaneously visualize time-varying multichannel EEG data.

In PCPs, the vertical direction corresponds to the number of items, while the horizontal direction represents the number of data dimensions. The number of available vertical pixels in the display space sets the upper limit for distinguishing values in each dimension. When using parallel coordinates to visualize high-dimensional data, several challenges can arise due to an excessive number of data items or dimensions. As for too many data items, lines representing data points can overlap excessively, making it difficult to discern individual patterns or trends. This overlap often results in a cluttered and unreadable visualization. Aggregating or down-sampling data points can help reduce the number of lines in the plot. Our previous work [9,10] summarizes these issues and proposes a pixel-aware approach [10] to reduce data items and represent an abstract version of the data to be efficiently transferred and rendered.

As the number of dimensions increases, each dimension adds a new axis, and PCPs become “visual clutter”. The greater the number of dimensions, the harder it becomes to identify meaningful patterns and relationships between variables. Additionally, rendering a large number of data items and dimensions can be computationally intensive, leading to slow performance and lag, particularly in interactive visualizations. One solution to these issues is the use of axis reordering, which arranges the most important dimensions in recognizable areas of the PCPs, improving clarity and usability.

Existing techniques for PCP axis reordering face several significant limitations. First, calculating metrics for axis reordering typically involves computing correlations and clustering between every pair of dimensions and optimizing axis arrangement. This process incurs heavy computational overhead, making it feasible only for datasets with tens of dimensions. For superhigh-dimensional data, these approaches become impractical. Second, previous methods largely overlook user interaction data, which is crucial for incorporating user-driven priorities and dimension selection. This hinders the system’s ability to adapt to user-specific goals and exploration patterns. Finally, complex or composite metrics for axis ordering reduce explainability, making it challenging for users to understand and effectively interact with the visualization.

Human-in-the-loop (HIL) interaction analysis, which incorporates expert know-how, is a preferable approach for exploring data through iterative trial and error. Andrienko et al. [11] explore a novel framework that integrates human expertise with machine learning to identify and classify behavioral patterns, particularly when precise pattern definitions are lacking. Although the framework is designed for time-series data, the broader HIL approach of using expert knowledge to define features and label examples for machine learning can be generalized.

Given the complexity of the semiconductor manufacturing process, this research is motivated by the need for interactive visual analysis to detect and address both known and unknown defects in the manufacturing process. Handling an explicit target/defect dimension within superhigh-dimensional data is a key aspect of the problem being addressed.

While dimension selection, data filtering, and down-sampling are widely used techniques for visualizing big data, interactively analyzing superhigh-dimensional sensor data to uncover defect root causes remains a challenge. Popular visualization platforms, including Tableau [12], Spotfire [13], and Polyspector [9], do not efficiently handle the interactive visualization of such high-dimensional data, particularly in the context of root cause analysis in semiconductor manufacturing lines.

Focusing on the HIL paradigm, we designed SPDC (Superhigh-Dimensional Parallel Coordinate) to address the challenges of analyzing defect causality within yield fields in semiconductor production lines. SPDC overcomes the limitations of existing interactive visual analysis methods for superhigh-dimensional datasets. In the HIL paradigm, user interactions take precedence over the importance scores of dimensions, enabling seamless integration of domain expertise into the analysis process. This approach enhances the interpretability, efficiency, and precision of defect analysis.

In the typical scenario for visual exploratory analysis of semiconductor production line sensor data, a user designates a target dimension that contains defect data. The user then interactively defines filtering ranges across multiple dimensions. These filters guide the selection of relevant dimensions and data items, which are subsequently down-sampled. This process substantially reduces the volume of data to be visualized, enabling effective and efficient interactive analysis.

In the context of SPDC applied to causality analysis, the explicit target or defect dimension allows for more efficient axis reordering computation compared to general assumptions in PCPs. To further enhance performance for visualizing large volumes of data, SPDC employs a server–client architecture. In this design, computationally intensive tasks, such as correlation analysis and down-sampling, are executed on the server side, ensuring efficient data handling while providing a responsive and interactive user experience.

Our contributions primarily include the following:

- implement an SPDC HIL paradigm using server–client architecture with on-demand server-side processing to enhance performance and responsiveness.
- enable efficient axis ordering for interactive visualization by leveraging the explicit target/defect dimension tailored for root cause analysis in the visual exploratory analysis of semiconductor production line sensor data.
- addressing inefficiency in interactive visual analysis for superhigh-dimensional datasets, particularly those involving tens of thousands of dimensions in semiconductor production lines.

In the following section, we discuss two topics of related work: the server–client architecture for big data visualization and axis reordering techniques for visual analysis. Section 3 outlines the visual exploratory analysis in a semiconductor manufacturing context, especially an SPDC server–client architecture within the HIL paradigm. Section 4 describes our method for interactive visualization, with an emphasis on updating flow and selecting visible dimensions. Section 5 provides details of server-side data processing, followed by implementation in Section 6. Section 7 evaluates the system with an ablation study, usability survey, and user interviews. Finally, we conclude with key insights and propose potential directions for future work.

## 2. Related Works

Handling large datasets with high dimensionality and an excessive number of items presents significant challenges. Storing all of the data locally for real-time rendering is often infeasible due to computational and memory limitations. Additionally, the sheer number of dimensions can result in “visual clutter” in line-based parallel coordinate representations, which hinder effective analysis.

Previous works with parallel coordinates have primarily focused on two aspects: utilizing server–client architectures to offload compute-intensive tasks to the server and leveraging pre-computed or layered data for visualizing large datasets; and employing axis ordering to emphasize important dimensions. This section explores two key areas of related work: server–client architectures for big data visualization and axis reordering techniques for visual exploration.

### 2.1. Server–Client Architecture in Big Data Visualization

Server–client architectures are integral to big data visualization, enabling efficient data processing and interactive user experiences. Recent research has explored their advantages and limitations. Cedilnik et al. [14] discuss the integration of a data server, render server, and client model within the ParaView framework to address challenges in remote visualization of large datasets. Similarly, Kruchten et al. proposed VegaFusion [15] to enhance scalability by performing server-side processing for large datasets, avoiding browser memory constraints and significantly improving performance. While both discuss handling large-scale data, they do not explicitly delve into high-dimensional data. Moritz and Heer [16] introduced automated techniques to partition computational workloads between server and client, optimizing visualization plans to minimize latency. The paper explicitly considers high-dimensional datasets as part of their optimization framework, but dimensionality reduction methods (e.g., PCA or t-SNE) require complex computation and are challenging for high-dimensional data.

In the context of Parallel Coordinate Plots (PCPs), Richer et al. [17] proposed HiePaCo, a focus+context representation combined with distributed computation. This approach leverages server-side scalability to maintain interactive responsiveness through a sophisticated client–server architecture featuring distributed computing and hierarchical aggregation. However, this method can be challenging to implement and demands substantial computational resources. Similarly, Cui et al. [18] developed a web-based confluent drawing method for PCPs, which employs binning-based clustering to mitigate visual clutter and overplotting. However, introducing confluent drawings may affect the usability and interpretability of visualizations, potentially limiting their effectiveness in certain use cases.

While these server–client architectures effectively manage large datasets with extensive data items or data dimensions, they often introduce additional system complexity and struggle with extremely high-dimensional data. Such approaches typically fail to scale for superhigh-dimensional datasets due to limitations in interactively reducing dimensions through visualization mechanisms. Addressing these challenges is the primary focus of this paper.

### 2.2. Axes Reordering for Visual Exploration

Axes reordering based on correlation, clustering, or common metrics [19] in PCPs is a pivotal technique for mitigating “visual clutter” in high-dimensional data visualization. These methods detect the properties by analyzing every pair of dimensions in the dataset and then determining the optimal axis arrangement. By strategically arranging axes, one can uncover hidden patterns, reduce visual clutter, and improve the interpretability of complex datasets. Recent research has introduced various methodologies to optimize axis ordering in PCPs.

Tran and Linsen [20] developed a method for automating dimension reordering and spacing in PCPs using Pearson’s correlation for unclassified data and class distance consistency for classified data. While this approach enhances visualization effectiveness, it is tailored to biological datasets and may not generalize well to other domains with different data structures.

Tilouche and Bassetto [21] formulated a binary optimization problem for coordinate ordering, proposing a computationally efficient greedy approach suitable for high-dimensional data. This method improved cluster detection and attribute dependency visualization, though binary optimization remained resource-intensive and limited in interpretability.

Lu et al. [22] introduced DACP, an arc-based visualization approach utilizing dimension-based bundling to mitigate clutter. They proposed two reordering techniques: a Contribution-Based Method that uses singular value decomposition (SVD) to rank dimensions by contribution scores and a Similarity-Based Method combining non-linear correlation coefficient (NCC) with SVD to arrange axes based on inter-dimensional correlations. These methods effectively reduce clutter but may confuse users unfamiliar with non-linear visualizations.

Tyagi et al. [23] developed PC-Expo, a real-time visual analytics framework enabling users to optimize axis ordering through the selection and weighting of various properties. This HIL approach supported flexible and interactive reordering to accommodate multiple analysis tasks. While PC-Expo is effective for datasets with tens of dimensions, its scalability becomes a significant challenge as the dimensionality increases.

Consequently, applying parallel coordinates to superhigh-dimensional data becomes impractical due to performance and usability constraints. Most prior works are effective only for datasets with tens of dimensions or pre-computed data, as the complexity of clustering and importance score computations limit scalability. Furthermore, the use of complex or composite metrics often introduces interpretation challenges, making it difficult for users to derive actionable insights. These gaps underscore the need for a system that combines scalability, interactivity, and data integrity preservation.

Given the complexity of patterns that can emerge in high-dimensional (HD) datasets, there is no single solution that universally fits all scenarios for PCP axis arrangement and analysis. Different use cases require conveying distinct insights through PCPs, which necessitates flexible and context-specific axis arrangements. User interactions play a crucial role in determining the order of dimensions, yet this factor is often overlooked in most existing methods.

In our target use case, which involves detecting defect causes in superhigh-dimensional sensor data, it is essential to explore raw data to preserve the understandability and interpretability of visual representations. This necessitates an efficient method for dimension selecting and axis reordering, an efficient trial-and-error approach for interactive visual analysis, and a consistent mechanism for incorporating user expertise into axis arrangements. Addressing these challenges forms the core focus of our paper.

## 3. Visual Exploratory Analytics in Semiconductor Manufacturing Context

The semiconductor manufacturing process is highly complex, with each product undergoing thousands of steps using a wide array of machine types and product lines, intricate process routes, and the dynamic nature of the manufacturing system. The lead time can be several months or longer. Due to this complexity and extended lead time, our target manufacturing system utilizes a private cloud to collect and integrate data corresponding to dies, wafers, and lots. Additionally, server–client architecture is employed for defect detection based on HIL analysis.

### 3.1. Integrating Sensor Data into Private Cloud

Data are continuously collected from sensors fitted to equipment or facilities and sent to the data analysis system in a private cloud. However, different sensors require different protocols. Therefore, an IoT gateway is employed to provide a unified solution for integrating various types of sensor data. The integrated data are used to identify clusters and association rules, pinpointing specific process conditions or equipment states that lead to defects and yield loss. The private cloud environment allows full control and ensures low data latency while benefiting from cloud features such as high availability, elasticity, and agility. Our SDPC system efficiently handles integrated sensor data from semiconductor production lines by identifying dies, wafers, and lots. As described in the following sections, the SPDC’s server–client architecture consumes minimal data to interactively visualize sensor data.

### 3.2. Server–Client Architecture Tailored for HIL Analysis

For superhigh-dimensional data, making all dimensions visible simultaneously is impractical due to limitations in display size and visual acuity. Our goal is to provide a general interactive visual analysis system that dynamically abstracts and purposefully displays the desired dimensions in a smooth and efficient manner.

**Figure 1:**
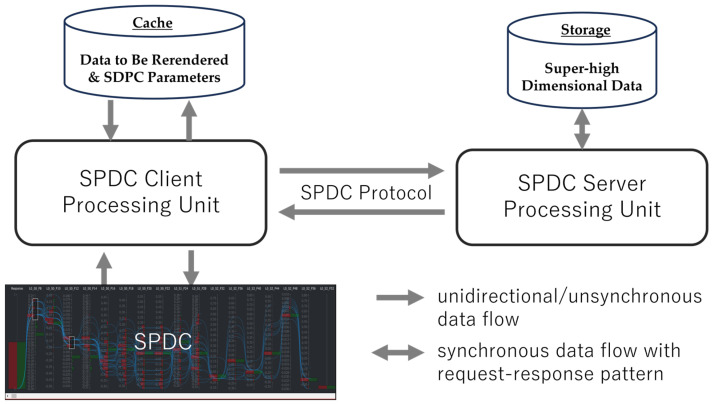
The HIL server–client architecture used in SDPC. The server processing unit handles computationally intensive tasks like importance score calculation, filtering, and down-sampling, while the client processing unit focuses on rendering and user interaction. This division ensures real-time responsiveness and efficient handling of superhigh-dimensional datasets. The SPDC protocol between the server processing unit and the client processing unit assumes the dimensions are ordered.

A critical requirement for interactive visual analysis is that users should not experience noticeable response latency caused by remote processing or data transfer. Based on the original Polyspector [9], we tailor the server–client architecture, as illustrated in Figure 1. This design accommodates the compute-intensive nature of the analysis and the data volumes distributed along horizontal and/or vertical dimensions. The server’s computational capabilities are leveraged to perform necessary computations for data reduction, tailored to meet the demands of instant user interactions. The reduced data are then transmitted and rendered within a web browser, even on a thin-client computer, ensuring a responsive and seamless user experience.

In superhigh-dimensional data, the combinations of values across dimensions grow exponentially, making it increasingly challenging to efficiently reduce the number of data items. This exponential growth often leads to significant “visual clutter” and performance degradation due to delays in data transfer and rendering. In a server–client architecture, users can freely select and explore data in any dimension as if the entire dataset were locally cached. By limiting the number of dimensions that need to be processed, transferred, and rendered the server–client architecture effectively mitigates the challenges posed by the “curse of dimensionality”.

SDPC is integrated into Polyspector [9], a general visualization platform designed to create built-in interactive visualizations with a server–client architecture [24]. It features loose coupling between the client and server; the only coupling between them pertains to a specific data source and its corresponding filter conditions. To support superhigh-dimensional data, additional SPDC protocol assumes that both the client and server are aware that the dimensions are ordered.

SPDC protocol is utilized to transfer requests and responses between client and server, enabling the server processing unit to efficiently identify visible dimensional data. The SPDC parameters—including the current dimension positions, visible dimension size, and total size of dimensions—are accessible to users in SPDC to facilitate visual analysis. As shown in Figure 1, SPDC’s server–client architecture integrates the SPDC plot, SPDC parameters, the client processing unit, and the server processing unit. This architecture employs a data reduction mechanism to address the challenges of superhigh-dimensional data by managing the dimensions and data volume transferred and rendered on demand.

The process begins with data extraction from storage based on user-defined filter conditions. The extracted subset typically contains superhigh dimensions. Importance scores are then calculated between the target dimension and other dimensions, creating an ordered list. Using this ordered list, a reduced dataset containing the most important dimensions is generated and further down-sampled to fit within the SDPC area. The down-sampled data are transferred to the client, cached, and rendered within the designated SDPC area. By reducing both the data items and dimensions rendered on the client side, this approach effectively mitigates issues related to data transfer delays, memory consumption, rendering performance, and visual clutter. The filter conditions and the SDPC area size, determined by the client, are key parameters sent to the server to facilitate this process.

In the HIL paradigm, user interactions involve two loops: an external loop for data-related interactions and an internal loop for appearance-related interactions.

- The internal loop operates on locally cached data and is triggered by actions such as adjusting chart designs and changing the color scheme. These interactions are processed entirely on the client side.
- The external loop, by contrast, handles data-related updates and is triggered by operations like modifying filter conditions for dimensions.

The external loop plays a critical role in data reduction, enabling interactive visual analysis. SDPC updating is initiated by user interactions and propagated through the external loop.

The mechanisms for interactive visualization of the external loop will be discussed in detail in the next section.

## 4. Interactive Visualization

**Figure 2:**
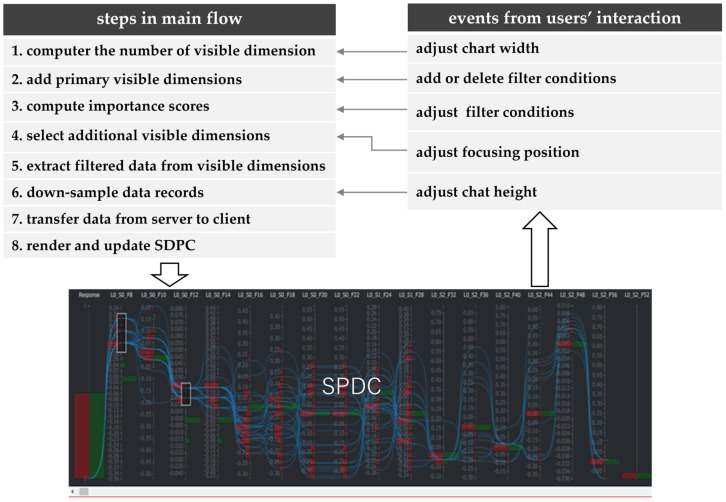
The interactive visualization flow within SDPC. The “main flow” manages data processing tasks, including dimension selection, data extraction, and down-sampling, while the external loop handles data-related updates. This flow ensures seamless updates to the visualization in response to user interactions. The SPDC snapshot is further explained in Figure 10.

The interactive visualization flow corresponds to the external updating loop, as shown in Figure 2. Dimension selection, dynamic filtering, down-sampling, and axis reordering are integral components of this loop.

By incorporating an HIL paradigm, SDPC extends the utility of PCPs, enabling users to iteratively refine visualizations through dimension selection, dynamic filtering, axis reordering, and real-time down-sampling. These features address critical challenges in semiconductor production, where identifying defect causality requires preserving and exploring relationships across tens of thousands of sensor dimensions in real time.

**Figure 3:**
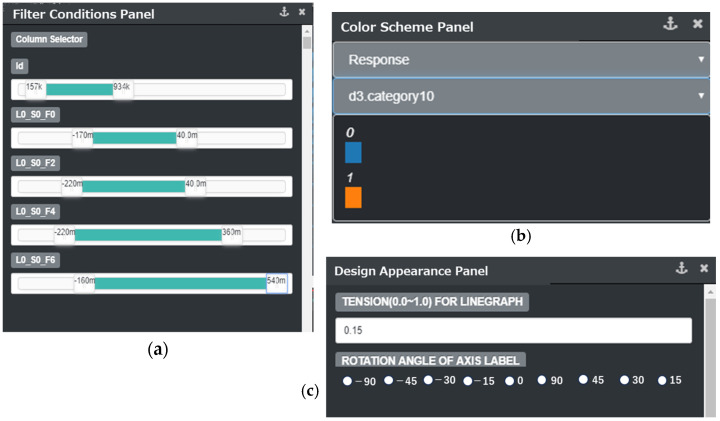
Examples of SDPC’s user interface for appearance and data-related interactions: (**a**) adjusting filter conditions across multiple dimensions; (**b**) customizing the color scheme for SDPC plot; and (**c**) modifying appearance parameters like axis spacing and chart size.

On the left-top of Figure 2 is the “main flow”, which includes the following steps:

1. compute the number of visible dimensions
  The number of visible dimensions is calculated based on the current SDPC area width and the user-specified interval between SDPC axes.
2. add primary visible dimensions
  Primary visible dimensions include three types:
  - color dimension: This is a virtual data dimension that helps define the color scheme and enables interactive exploration of corresponding data items. A color axis uses a specified color scheme. Figure 3b shows the panel to set the color scheme for the color dimension.
  - filter dimensions: Once a filter condition is set, that dimension is considered important by the user. This is the reason that dimensions with filter conditions should be included in the visible dimensions. Filter conditions can be configured either interactively or offline.
  - target dimension: The target dimension is the user-selected dimension for investigating defects. It should be a visible dimension, and generally, the target dimension is also used as the color dimension.
3. compute importance scores
  Importance scores between the target dimension and other dimensions are computed based on the current filter conditions and the target dimension.
4. select additional visible dimensions
  A list of additional visible dimensions is extracted by sorting all dimensions in descending order of their importance scores, and the most important dimensions not included in the primary visible dimensions are selected.
5. extract data from visible dimensions with filter conditions
  Data corresponding to all visible dimensions are extracted from the original dataset based on the filter conditions.
6. down-sample data items
  The number of data items is reduced through down-sampling according to the current height of SDPC axes.
7. transfer results from server to client
  The down-sampled data are transferred to the client and cached for usage in the internal loop.
8. render and update SDPC plot
  The cached data are rendered to update the SDPC plot. The target dimension is displayed as the first visible dimension on the far left.

**Figure 4:**
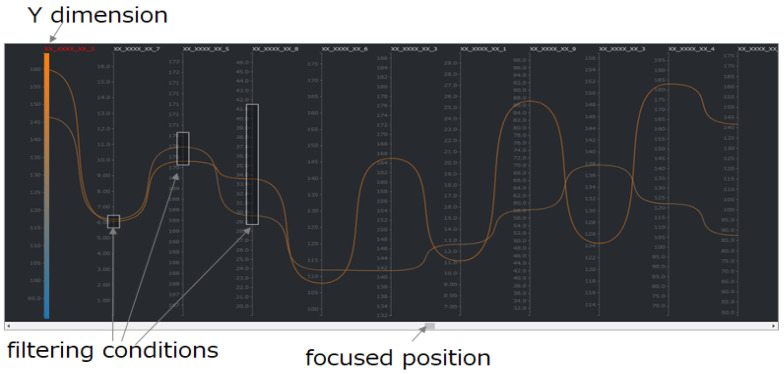
Example of setting filter conditions directly on visible axes in SDPC. Filtered dimensions remain visible regardless of their importance score or slider bar adjustments, allowing users to iteratively refine their focus during defect analysis.

**Figure 5:**
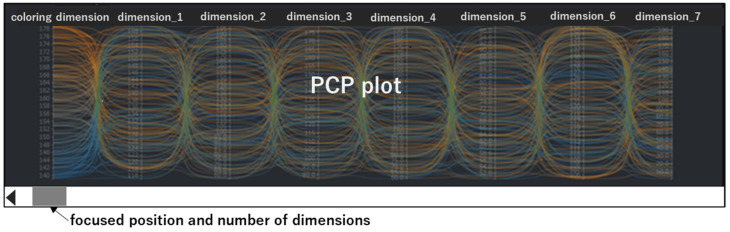
Integrated slider bar arrangement within the SPDC plot. The slider represents the total number of dimensions in the dataset and allows users to select a specific range of dimensions for dynamic visualization. The different colors of lines represent various values in coloring dimension.

After the initial SDPC display, a user interaction event on the SDPC plot is sent to the server, triggering the corresponding processing steps in the “main flow” and ultimately updating the SPDC plot. The types of user operation events, illustrated on the right side of Figure 4, include the following:

- adjust chart width
  This event feeds back to step 1 in the “main flow”, triggering recalculation of the number of visible dimensions and subsequent steps.
- add or delete filter conditions:
  This event feeds back to step 2 in the “main flow”, triggering recalculation of the primary visible dimensions and subsequent steps.
- adjust filter conditions
  This event feeds back to step 3 in the “main flow”, triggering a recalculation of additional visible dimensions and initiating subsequent steps. Similarly, changing the target dimension or modifying the algorithm used to compute importance scores produces the same effect.
- adjust focused position in the dimension ordering list
  This event feeds back to step 3 in the “main flow”, triggering the recalculation of importance scores and subsequent steps. One of these subsequent steps is selecting additional visible dimensions in step 4. Additional visible dimensions are indicated by the knob on a slider bar, as shown in Figure 3 or Figure 5.
- adjust the chart height
  This event feeds back to step 6 in the “main flow”, initiating down-sampling of the data and subsequent steps.

### 4.1. Select Dimensions

#### 4.1.1. Select Dimensions by Filter Conditions

Filter conditions are essential for interactive visual analysis. In a traditional parallel coordinates plot, all axes are visible, allowing users to interactively specify or update filter conditions for any dimension. In SDPC, however, only a small subset of dimensions is visible at a time. To enable users to update filter conditions while remaining aware of the filtering context, dimensions with active filter conditions are kept visible in the SDPC plot, even if they fall outside the focusing range of the ordered list.

Filter conditions can be set in two ways. One approach involves setting filters directly on SDPC axes, as shown in Figure 4, where three axes (dimensions) are displayed with filter conditions. This method is commonly used to experiment with filter settings and observe the resulting patterns in SDPC visualization. However, a drawback of this approach is that it does not allow filter conditions to be applied to axes that are not currently visible.

Another way to set filter conditions is through a separate “Filter Conditions Panel”, as illustrated in Figure 3a. In semiconductor production lines, experienced users typically possess prior knowledge of defect patterns and use this panel to verify whether these patterns correspond to a defect’s root cause. The panel allows users to conveniently retrieve specific dimensions by column names and apply experienced values as filter conditions.

Filter conditions applied in one user interface are updated in real time across other interfaces. Dimensions with filter conditions are counted as primary visible dimensions. Axes with filter conditions remain visible regardless of other factors, such as adjustments to the knob position on the slider bar, as described in the next section.

#### 4.1.2. Select Dimensions by Importance Scores

Importance scores are computed using filtered data. After applying filter conditions, the number of filtered data items is significantly smaller than the original dataset, enabling computations to be completed in real time, even for datasets containing billions of entries. Once the dimensions are ordered based on their importance scores, the most important dimensions—excluding the primary visible dimensions—are selected as additional visible dimensions. Assuming the total number of visible dimensions is N, and the number of primary visible dimensions is M, the number of additional visible dimensions is calculated as max(N−M,0). The value of 0 indicates that all visible dimensions are primary visible dimensions (i.e., those with filter conditions), eliminating the need to compute importance scores or select additional visible dimensions.

#### 4.1.3. Select Dimensions with Slider Bar

Within the SDPC plot, a slider bar is a necessary component and serves as a range selector and operation panel. The length of the slider bar represents the total number of dimensions in the dataset. The knob position on the slider bar indicates the starting position of the dimensions to be displayed, while the knob’s length corresponds to the number of additional visible dimensions.

Except for the primary visible dimensions with filter conditions and the target dimension, all other dimensions are ordered by their importance scores. This ensures that the knob’s position maintains a consistent interpretation, enabling users to readily identify current importance rankings. When adjusting the slider bar, the changing knob position is frequently sent to the server to infer additional visible dimensions and update the SDPC interactively. While moving the knob dynamically updates the additional visible dimensions, the primary visible dimensions always remain visible in the SDPC. Using the slider bar in the SDPC plot enables users to interactively browse the full range of dimensions without “visual clutter” in a parallel coordinates plot. To avoid triggering events too frequently when adjusting the slider bar with the mouse, the event is only triggered upon releasing the mouse button.

Two types of slider bar arrangements are used in SDPC plots to specify dimension positions in the ordered dimension list. The first arrangement embeds the slider bar directly within the SDPC plot, as shown in Figure 5.

**Figure 6:**
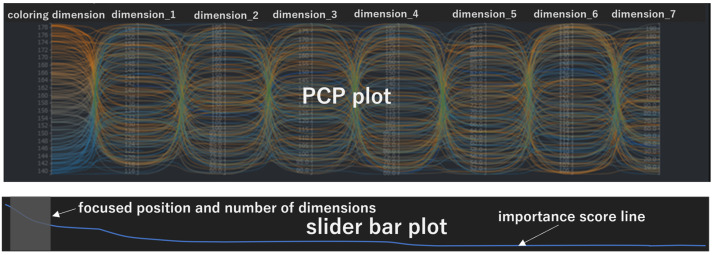
A separated slider bar arrangement with a linked SDPC plot. The slider bar at the bottom provides additional information, such as the importance scores of dimensions, enabling users to refine dimension selection while maintaining clarity in the main visualization.

The second arrangement of a slider bar, shown in Figure 6, separates the parallel coordinates plot from a slide bar plot, enabled to specify the dimension position. An operation event on the slide bar plot chart is data-linked to the parallel coordinates plot through an external mechanism, such as a server worker [10], to receive events from the slider bar plot and send received events to the parallel coordinates plot. This arrangement allows for greater flexibility in adjusting the layout, and the logic for linking the two plots and their corresponding data is more independent compared to the first layout. For example, the slider bar plot at the bottom of Figure 6 also shows importance scores corresponding to the visible dimensions, aligning with the SDPC at the top.

### 4.2. Visualizing Dimensions

For numeric or ordinal data onto multiple axes, each axis corresponds to a numerical variable, and data points are depicted as lines that connect these axes, with their positions reflecting the respective values. Examining interactions between lines across axes allows users to identify correlations, trends, and data ranges among various variables. For categorical data, axes feature discrete segments to group and compare patterns, enabling the observation of distributions within or between categories.

In SDPC visualization, special considerations are applied for categorical dimensions and dimensions with missing values and the target dimension, which are elaborated as follows.

#### 4.2.1. Categorical Dimensions

Visualizing categorical variables alongside numeric or ordinal variables is often desirable; however, parallel coordinates cannot directly render categorical variables. To address this, we transform categorical variables into ordinal variables using one of the following three methods.

- In the order of data items as they are

In many application scenarios of our root cause analysis context, the order of raw data items on the server holds significant meaning. Retaining this order and using the numeric index of each record provides a convenient method to convert a categorical dimension into an ordinal dimension.

- In the decent order of occurrences

A value’s frequency or number of occurrences is an important indicator of its significance. For categorical dimensions requiring such analysis, the system computes basic frequency statistics and appends them before sending the data to the client. This process allows the system to treat categorical dimensions as ordinal dimensions. Additionally, it can combine minor categories into an “Other” category to reduce the total number of categories and simplify visualization.

- Lexical order

Searching for values in a categorical dimension is intuitive for end users, particularly when they have domain knowledge. By combining text search filters with the lexical ordering of values within a categorical dimension, specific values can be efficiently located even within a large list.

#### 4.2.2. Dimensions with Missing Values

**Figure 7:**
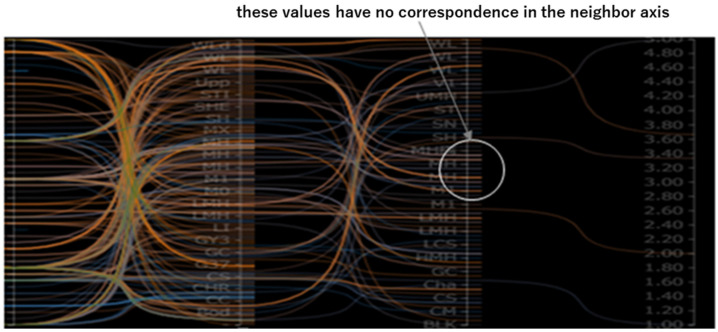
Representation of missing values in SDPC. Short lines along the axis highlight missing values in adjacent dimensions, preserving their distribution patterns without obscuring inherent data characteristics.

The positions and distributions of missing values provide crucial insights into the underlying characteristics of the data. To preserve these details, it is preferable to represent the status of missing values as they appear in the original dataset. Automatically filling these values using algorithms before visualization may obscure their inherent patterns and relationships. As demonstrated in Figure 7, the short lines along one axis represent the presence of missing values in the corresponding adjacent axis.

#### 4.2.3. Target Dimension

A target dimension serves as the dependent variable for evaluating the importance of other dimensions. In defect detection analysis within semiconductor manufacturing processes, the target dimension (Y dimension) is typically designated by the user to identify the root causes of defects in other dimensions. The correlation between the target dimension and other dimensions is computed separately, yielding importance scores.

From a visual analysis standpoint, the target dimension should always be displayed alongside other dimensions in an SDPC plot. This is because the target dimension, along with its filter conditions, functions as a fundamental indicator of “good” or “bad” outcomes based on the user’s expectations. Since a variable is naturally more strongly correlated with itself than with any other variable, it is logical to position the target dimension as the first dimension in descending order of correlation scores. Furthermore, using the target dimension as the coloring dimension is often advantageous, as the values in the coloring dimension determine the line graph colors according to a specified color scheme.

## 5. Real-Time Data Processing on the Server

In the client–server architecture described in the previous sections, which facilitates interactive dimension selection, data item extraction, and down-sampling, the performance of server-side processing is a crucial factor in enabling real-time interactive visual analysis of superhigh-dimensional data. By separating server-side processes from client-side rendering tasks, server-side computations can be distributed and parallelized. Simultaneously, algorithms and their parameters can be interactively adjusted through the client-side user interface. In server-side processing, the complementary relationship between dimension selection and down-sampling plays a crucial role in achieving effective data reduction. This section delves deeper into these processes, examining their interplay and significance.

As discussed in the previous section, the characteristics of the dataset—such as data types, the presence of missing values, and the number of data items—can vary significantly across cases. In semiconductor production, missing line data values frequently occur, and categorical values are also critical to analyze together with quantitative data. These factors are particularly considered when selecting algorithms to compute importance scores for all dimensions. Tree-based algorithms are preferred, as they effectively handle issues related to missing and categorical values.

While implementing SDPC, down-sampling becomes an essential consideration when the dataset exceeds thousands of data items. We employ an improved data cube down-sampling method to approximate the user’s desired sample size, ensuring interactive visual exploratory analysis with SDPC.

### 5.1. Dimension Importance Computation

Visible dimensions update instantly through user interactions; however, in previous works such as PC-Expo [23], axis reordering computations limited performance when handling high-dimensional data. The computation of importance scores poses a similar bottleneck in achieving interactive performance in SDPC. Fortunately, specifying the target dimension allows us to focus on the correlation between the target dimension and other dimensions for ordering purposes. While a direct method to calculate correlation involves assessing the relationship between the target dimension and other dimensions, tree-based models offer a more efficient alternative. These models require minimal preprocessing to handle missing or categorical values, making them well-suited for this task.

To ensure efficiency, we employ the following algorithms to compute importance scores on the server. Users can select the desired algorithm via the operation panel in the user interface, and the selection is transmitted to the server for executing dimension selection.

#### 5.1.1. Correlation Between Dimensions

When both dimensions are numeric, the Pearson correlation coefficient is used to calculate the correlation score. If one of the dimensions is of categorical type, the Spearman correlation coefficient is employed to compute the correlation score.

#### 5.1.2. Tree-Based Model for Importance Computation

For dimensions with a high percentage of missing values, imputing the missing data can be challenging. To address this, tree-based models such as Random Forest, LightGBM [25], and XGBoost [26] are utilized to compute importance scores. In this approach, the target dimension is treated as the dependent variable (Y), while the other dimensions are considered independent variables (X).

#### 5.1.3. Stacking Model to Merge Important Scores

We observed significant variations in the ranking of dimensions based on importance scores when switching between algorithms or adjusting their parameters. To achieve more stable results, a stacking model [27] is employed to assemble the outputs from multiple tree-based and correlation-based methods.

### 5.2. Down-Sampling

Down-sampling serves as an efficient approach to improve data transfer and rendering performance while maintaining visual clarity. However, random sampling, though computationally fast, risks removing critical data items and is non-repeatable, which can confuse users.

To overcome these limitations, we employ a more flexible down-sampling algorithm. This approach improves the adaptability and precision of the down-sampling process while maintaining interactivity.

**Figure 8:**
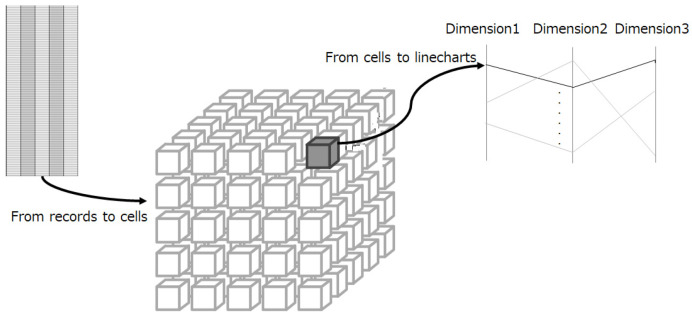
Down-sampling in SDPC. Data points are mapped to a multi-dimensional data cube, with only non-empty cells transferred for rendering. This approach significantly reduces the volume of data while maintaining the integrity of critical patterns.

As illustrated in Figure 8, data items are first mapped onto a data cube, with non-empty cells subsequently represented as line graphs in the parallel coordinates plot. Each cell in the data cube maintains a 0/1 value or count to indicate whether it is empty. Consequently, the number of resulting samples does not exceed the total number of cells.

To construct the data cube, the minimum number of bins for each dimension is estimated using the following formula:

(1) $$number\_of\_bins=Max(\sqrt[{k}]{R},\;c)$$

Here, k represents the number of visible dimensions, R denotes the number of items required by the client during an interaction updating loop, and c is a constant. Once the number of bins for each dimension is determined, all data are mapped into cells of the k-dimensional data cube.

**Figure 9:**
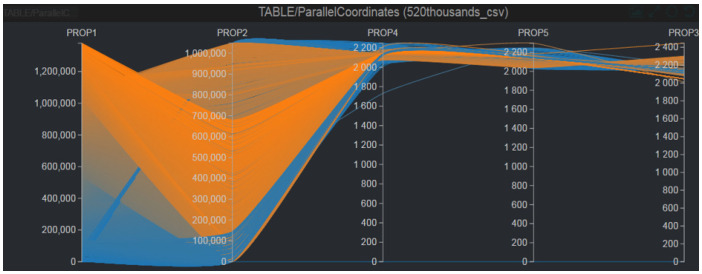
An example of down-sampling in SDPC. The original dataset with 520,000 items is reduced to 400 representative items, ensuring that critical outliers and patterns are preserved for effective visual analysis.

Given the sparsely distributed non-empty cells in the data cube, the number of bins in Formula (1) for each dimension can be adjusted using the constant c to balance appearance and performance. This adjustment ensures that salient data items in sparse regions are preserved while data items in overlapped or dense regions are significantly down-sampled. An example of the down-sampling process is depicted in Figure 9.

## 6. Implementation

The proposed SDPC system is implemented within Polyspector [9], a Big Data visualization platform. We have deployed the system and validated its effectiveness in interactive visual analysis with sensor data collected from the production lines in a semiconductor factory. The dataset contains tens of thousands of dimensions and approximately 100,000 data items, encompassing both categorical and numerical data types with varying percentages of missing values (ranging from 0 to 100%).

In a real-world environment, the objective of visual exploratory analysis is to confirm known causes and identify unknown causes of defects with respect to a specified response variable (the target dimension). Defects are represented by the response variable, with known causes linked to recognizable patterns in certain dimensions. SDPC facilitates exploring and confirming these patterns across multiple dimensions. Conversely, unknown causes correspond to defects where such patterns cannot be observed. By leveraging SDPC’s visualization and interaction capabilities, factors contributing to defects can be efficiently identified.

During experimentation, we used a Dell PowerEdge R930 server equipped with 144 Intel^®^ Xeon^®^ E7-8880 CPUs (2.30 GHz) and 2 TB of memory, achieving maximum response times of about 1 s. Factory engineers, accustomed to tools like Spotfire [13] and Tableau [12], highly evaluated SDPC’s performance in identifying patterns across dimensions related to manufacturing steps or equipment.

We demonstrate the SDPC implementation using the publicly available “Bosch Production Line Performance—Reduce Manufacturing Failure” dataset [28]. In the Bosch dataset, we merged numerical and categorical dimensions to observe their relationships. The merged dataset contains 3110 dimensions and 1,183,748 data items. The target dimension, “Response”, has binary values {0, 1}, treated as an ordinal variable. The “Id” column is retained after down-sampling to allow users to trace individual samples.

**Figure 10:**
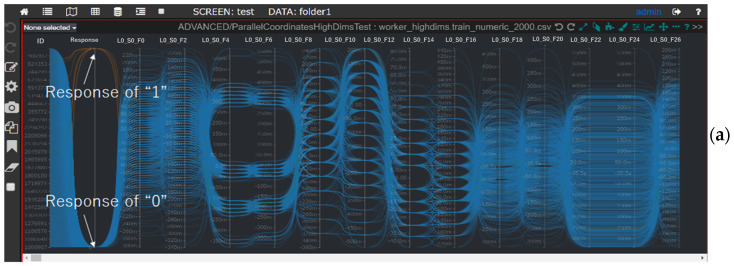

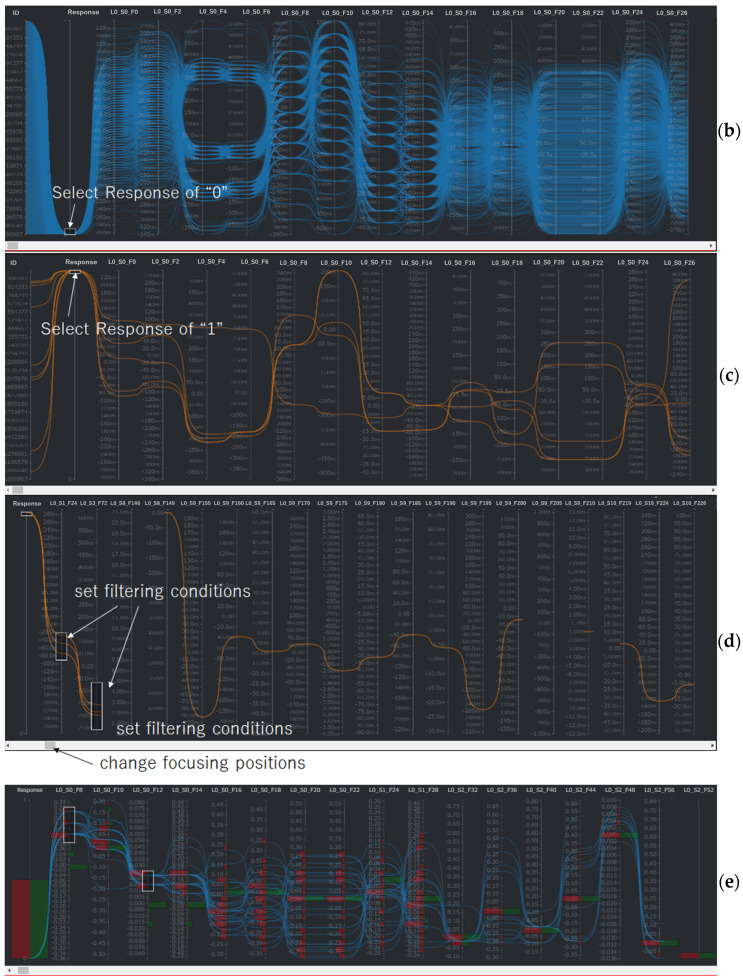
SDPC snapshots of the Bosch Production Line Performance dataset. The different colors of lines represent various values in coloring dimension (the far-left axis). (**a**) The initial visualization with “Response” as the target dimension; (**b**,**c**) highlighting normal and abnormal response categories across all dimensions; (**d**) applying filter conditions to focus on a subset of dimensions; and (**e**) overlaying histograms to compare data distributions before and after filtering, highlighting key patterns.

**Visualization insights (Figure 10)** include the following:

- **Figure 10a:** The “Id” dimension is uniformly distributed, reflecting its role as an index. Normal response values (“1”) are significantly more frequent than abnormal values (“0”).
- **Figure 10b:** The distribution of normal response values highlights typical ranges or patterns across dimensions.
- **Figure 10c:** The distribution of abnormal response values reveals potential defect causes inferred from patterns in non-target dimensions.
- **Figure 10d:** Applying filter conditions on “L0_S1_F24” and “L0_S3_F73” isolates a subset of interesting samples. Slider adjustments allow users to examine values in other dimensions. Categorical dimensions with lower importance scores, such as those filtered, appear farther from the target dimension “Response” in the SDPC plot.
- **Figure 10e:** Filtering conditions on “L0_S0_F8” and “L0_S0_F12” are predefined based on engineers’ experience to identify the root causes of defects (response “0”). The overlaid histograms suggest potential causes related to specific values, including 0.00 in “L0_S0_F20” and “L0_S0_F22”, −0.04 in “L0_S0_F24”, −0.03 and −0.13 in “L0_S0_F28”, and 0.01 in “L0_S0_F32”.

In SPDCs, the system can display additional information in two ways. The first approach uses linked charts [10], such as the slider bar plot shown in Figure 6. The second approach overlays histogram plots on the PCP, as illustrated in Figure 10e. Since line overlaying is minimized through down-sampling, histograms are added to the SPDC plot to enhance usability. These overlaid histograms allow users to compare data distributions before and after filtering, ensuring a comprehensive understanding of the data while maintaining interactivity and efficiency.

## 7. Evaluation

We have enhanced Polyspector [9] with SDPC functionality for over six months to aid in initially identifying defect causes in semiconductor production lines. During this period, an engineer used SDPC two to four times per week to analyze sensor data collected from semiconductor production equipment and environments. SDPC visual analysis serves as an initial step in the root cause analysis process. After identifying candidate dimensions through SDPC visual analysis, traditional methods such as data cleaning and machine learning are applied to confirm the findings.

Compared to previous visual analyses, which require engineers to repeatedly select and explore dimension groups, SDPC streamlines the process by interactively analyzing all dimensions at once. This approach makes it easier to identify relationships and pinpoint defect causes. Based on practical engineering data, SDPC reduces visual analysis time by two-thirds—cutting it from six hours to just two hours per week per person.

This section presents additional evaluations of SDPC, focusing on its effectiveness and efficiency. These assessments include a performance ablation study, a system usability survey, and user interviews. The usability survey focuses on the system’s performance in handling superhigh-dimensional data, measuring response times, and assessing the intuitiveness of its interface. Meanwhile, user interviews provide qualitative insights into the experiences of participants, highlighting the strengths of SDPC in interactive visual analysis and identifying potential areas for improvement.

The evaluation aims to determine how effectively SDPC supports defect detection and causality analysis in superhigh-dimensional datasets from real semiconductor production lines. It examines the system’s ability to enhance data exploration, recognize patterns, and integrate user feedback. Additionally, this section explores key aspects such as ease of use, creativity, and the integration of visual analysis with other analytical methods.

### 7.1. Performance Ablation Study

The Polyspector [9] platform’s server–client architecture offloads intensive computation to the server. In Table 1, we compare the functionalities of the server–client architecture across different implementations: PCPs in Tableau [12], PCPs in Spotfire [13], PCPs in Polyspector [9], and SDPC. All these implementations support updating visualizations with interactive operations. Tableau [12] and Spotfire [13] recommend pre-aggregating data for datasets with a large number of rows to reduce data items. Without customizing the server or client processing unit, as in SPDC, both are inefficient for superhigh-dimensional data. In contrast, Polyspector [9] utilizes real-time down-sampling but is inefficient for superhigh-dimensional data. With the current implementation of SDPC in Polyspector [9], enhanced down-sampling mechanism and optimized SDPC data transfer protocol enable interactive visualization of superhigh-dimensional data.

**Figure 11:**
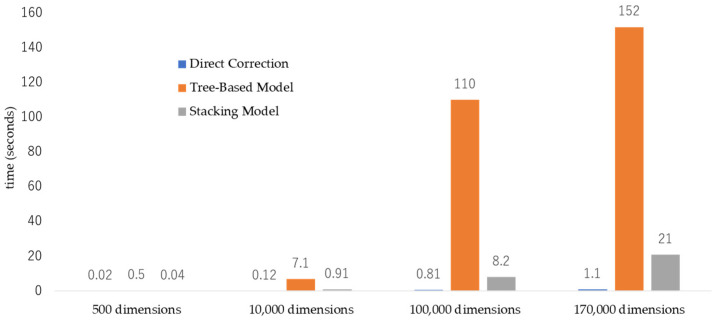
Comparison of performance to show 10 visible dimensions.

To demonstrate the feasibility of the proposed method, we first conducted an ablation study to evaluate the performance of axis ordering algorithms. In Figure 11, the server-side performance of axis/dimension ordering is compared across three algorithms: direct correlation, tree-based model, and stacking model. This comparison uses a real dataset comprising 13,000 items (product serial numbers) and 170,000 dimensions from semiconductor manufacturing lines.

When the number of dimensions increases from 500 to 170,000, axis ordering using direct correlation demonstrates the best performance, achieving computation times of less than 1.1 s across all cases. Axis ordering with the stacking model shows the second-best performance, with computation times exceeding 1 s when the number of dimensions surpasses 10,000 to 11,000. In contrast, axis ordering using a tree-based model is only suitable for datasets with fewer than 1000 dimensions, as its performance significantly degrades with higher dimensionality.

In addition to axis ordering, another computation-intensive process in the “main flow” of the server–client architecture is down-sampling. Additionally, a significant portion of time is consumed during the initialization of the server processing unit when the data source is switched by the user within the SPDC plot. Table 2 summarizes the response times measured from the moment an operation is triggered to the point when the SPDC plot is updated. The evaluation was conducted using the same hardware environment and dataset as the previous ablation study, with all filtering conditions cleared to avoid interference in time estimation. The number of visible dimensions was fixed at 10.

The time required for the initial SPDC visualization is approximately 10 s, primarily due to data loading and server-side initialization. Subsequent rendering times range between 2 and 3 s, encompassing processes such as axis ordering, down-sampling, and others. Given that axis ordering takes roughly 1.1 s and that the time required for other processes is significantly less than that for axis ordering and down-sampling, the estimated down-sampling time for this case (130,000 items and 10 visible dimensions) is approximately 1.0–1.5 s. Increasing the number of visible dimensions proportionally extends the down-sampling time.

As shown in Table 2, SPDC with an integrated slider bar is more efficient than SPDC with a linked slider bar chart; however, the difference is negligible compared to the overall suitability of the user interface. According to our user interviews, the request-response time was deemed acceptable by engineers working in semiconductor manufacturing sites. The current SPDC performance can be further enhanced by adding more CPU cores or leveraging GPU acceleration, which highlights another advantage of the server–client architecture.

### 7.2. System Usability Survey

The participants in the survey were deliberately selected to ensure diverse and relevant expertise. They included expert engineers with extensive experience in semiconductor production lines and researchers with over two years of experience in information visualization. Their diverse backgrounds provided a balanced perspective on SDPC’s usability and effectiveness in real-world applications.

The survey involved ten participants, including eight production line engineers and two information visualization researchers. The researchers used the system with production line data to familiarize themselves with SDPC functionality and to perform various causality analysis tasks.

Every participant was interviewed to collect feedback on the usability of SDPC. This survey aimed at evaluating SDPC for multiple factors based on the system usability scale (SUS) [29]. The SUS score is an industry standard for quantifying the usability of any visual analytics tool through a series of questions that focus on evaluating different aspects of the tool and that are sequenced alternatively to focus on positive and negative aspects. Participants are required to answer each question on a 5-point Likert scale that ranges from Strongly Disagree (1) to Strongly Agree (5).

#### Results

The SUS questionnaire and the average scores for all participants are presented in Table 3. For odd-numbered questions (Q1, Q3, Q5, Q7, Q9), higher scores indicate more positive feedback. Conversely, for even-numbered questions (Q2, Q4, Q6, Q8, Q10), lower scores reflect more positive feedback.

The SUS scores for SDPC ranged from 73 to 85, with an average score of 78. This score indicates good usability, exceeding the industry benchmark of 68 for visual analytics tools. Participants highlighted SDPC’s usability, attributing it to its ability to handle high-dimensional data without significant response delays.

Further analysis of the participants’ responses to individual SUS questions (see Table 3) revealed that the question receiving the most positive usability feedback was (Q2): “*I found the system unnecessarily complex*”. Similarly, questions (Q1), (Q4), and (Q9) also received favorable responses. These results suggest that SDPC is easy to use, and users feel confident in the results it generates. This feedback aligns well with the fundamental principle of explainability that guided the development of the tool.

These findings demonstrate that SDPC effectively supports real-time and accurate visual analysis to detect defect causes in semiconductor production lines. Both expert engineers and researchers reported similar positive experiences with SDPC, indicating that the tool is achieving its primary goals across diverse user groups.

Despite the predominantly positive feedback, (Q6): “*I thought there was too much inconsistency in this system*” and (Q8): “*I found the system very cumbersome to use*” received mixed reviews. These responses suggest that certain detailed features require improvement to reduce users’ workload and enhance the overall experience. To better understand these concerns, a follow-up interview session was conducted, as discussed in the user interviews section.

### 7.3. User Interviews

We selected two production line engineers (E1 and E2) and one information visualization researcher (R1) for interviews. These interviews helped us gain further insights into user experience with SDPC, its limitations, and potential areas of improvement. Some of the interview results and user comments are discussed below. We have categorized the user comments based on their experience with SDPC.

Effectiveness in identifying defect causes: All participants agreed that the fast response of SDPC for interactive visualization is highly useful for exploring related dimensions and ranges in high-dimensional data. Both E1 and E2, who have prior experience with automated machine learning analysis and BI tools, such as Tableau [12] and Spotfire [13], highlighted that previous systems were unable to handle such high-dimensional data interactively. For them, SPDC was their first experience of interactively exploring this type of data. Participant R1 remarked, “*Showing all dimensions with filter conditions is really helpful. Sometimes, I set up experimental ranges and quickly find the root causes I want to identify*”.

R1 suggested an improvement of the timing to trigger SPDC updating, commenting, “*Sometimes I want to fix the axes and their orders when adjusting filter conditions. The frequent updating of axes and their ordering causes some important hints to be lost*”. Based on this feedback, we have incorporated an option to temporarily fix axes and their ordering during user operations to improve usability.

Usability comparison with machine learning methods: E1 and E2, who have experience using machine learning methods for causality analysis, observed that different use cases often require distinct prior knowledge. They noted that the dynamic nature of production lines makes it challenging to collect all raw data in a timely manner, which often makes visual analysis a more efficient approach. SDPC enables users to interactively explore all raw data, allowing them to efficiently and promptly identify fixed patterns. Once these patterns are identified, machine learning methods can be applied routinely for further analysis. With an explicit target dimension, causality analysis with SPDC can be seamlessly combined with supervised machine learning. R1 suggested integrating visual analysis with machine learning patterns for a more comprehensive approach. However, he acknowledged that achieving a seamless integration would require further research and development.

Ease of usage and creativity: Participants highlighted the intuitive nature of SDPC for exploring high-dimensional data. E1 and E2 emphasized that the system’s ability to display raw data interactively significantly enhanced their understanding of defect causality, enabling them to easily spot patterns that might otherwise go unnoticed. They found the interface straightforward to use, even for complex datasets, and appreciated the dynamic features such as filtering and reordering dimensions.

R1 noted that SDPC encouraged creative problem-solving by allowing users to experiment with different views and filter conditions interactively. He remarked that this flexibility fostered new insights, particularly in scenarios where domain knowledge needed to be combined with raw data exploration. However, he suggested further improvements to enhance user creativity, such as offering customizable visualization templates and enabling better integration with external analysis tools.

All participants agreed that SDPC effectively bridged the gap between data exploration and decision-making, making it both an easy-to-use and powerful tool for high-dimensional data analysis.

## 8. Conclusions and Future Work

This paper addresses the challenges of interactive causality analysis for superhigh-dimensional datasets in semiconductor production lines. Given the complexity of the semiconductor manufacturing process, each product passes through thousands of operations over a long period of production cycle, generating vast amounts of sensor and environment data. For each product, the collected data can span tens of thousands of dimensions. We have highlighted that the system is specifically designed for causality analysis, where handling an explicit target dimension within superhigh-dimensional data is a key aspect of the problem being addressed.

By integrating an HIL paradigm, we developed a scalable approach to managing large data volumes and interactions. This was achieved through a tailored server–client architecture, dynamic data reduction, and intuitive visualization methods. The use of filter conditions and importance scores to select dimensions and dynamically update visible axes of parallel coordinates ensures that both raw data exploration and expert-driven analysis remain interpretable and actionable. SPDC enables real-time interaction, significantly reducing delays and visual clutter while maintaining the visibility of critical dimensions. By visualizing integrated sensor data through SPDC, our system enhances defect detection and optimizes semiconductor production. Integrating advanced visual insights and domain expertise reduces visual analysis time by two-thirds—cutting the process from six hours to just two hours per week for each on-site engineer. This optimization boosts operational efficiency and increases yield rates, leading to more effective production processes.

We observe that Python can be integrated into the server processing unit in Tableau [12] from version 10.1, and both R and Python can be similarly integrated in Spotfire [13] from version 10.7. Furthermore, Tableau [12] and Spotfire [13] offer functionality to transfer filter conditions between charts. As we implement SPDC in Polyspector [9], we plan to extend its capabilities to Tableau [12] and Spotfire [13].

While the system demonstrates significant and irreplaceable strengths in visualizing superhigh-dimensional data, its limitations offer valuable opportunities for future research. One key area for improvement is the integration of text semantic retrieval with the axes ordering. In semiconductor production lines, sensor data are often accompanied by manually input data tags or comments that contain critical knowledge for analyzing defect causes. Leveraging this textual information could significantly enhance the analysis process. Refining the axis reordering mechanism to incorporate long-term user feedback could further improve the system’s flexibility and usability.

Another future direction involves integrating machine learning techniques to better align with user-defined priorities and patterns. Frequently, identifying unknown defects in the manufacturing process is as critical as determining the root causes of known defects through interactive visual analysis. Domain-specific insights from engineers might lead to focusing only on known defect patterns, potentially overlooking novel or emerging anomalies. Integrating unsupervised learning methods (e.g., clustering, dimensionality reduction) to suggest initial dimensions or clusters for exploration would ensure that the user does not overlook important data aspects. Uncovering unknown defects requires a combination of machine learning and visual analysis, used alternately, to effectively uncover and understand these anomalies.

We believe visual causality analysis with superhigh-dimension data has a broader context. Expanding the system’s applicability to fields beyond semiconductor production lines, such as bioinformatics or financial analytics, represents a promising avenue for exploration. Additionally, evaluating the system in more diverse real-world applications and conducting user studies will provide valuable insights for refining its functionality and usability.

Through these enhancements, the system can serve as a robust tool for interactive visual analysis, driving efficiency and insight across various domains handling complex, high-dimensional data.

## Figures and Tables

**Table 1:** Functionality comparison of PCP implementations across different platforms. The Symbol 〇 indicates the feature is full supported, △ indicates the feature is partially suppored, and ×indicates the feature is bot suppored.

	PCPs in Tableau with Server-Client Architecture	PCPs in Spotfire with Server-Client Architecture	PCPs in Polyspector	SPDC
updating with interactive operations	〇	〇	〇	〇
real-time down-sampling	△	△	〇	〇
superhigh-dimensional data interactive visualization	×	×	×	〇

**Table 2:** Average response time of SPDC.

	PCPs in Polyspector	SPDC with Linked slider Bar Chat	SPDC with Integrated Slider Bar
request-response time for 1st rendering	11 s	11 s	11 s
request-response time for subsequent rendering	-	2.5 s	2.3 s

**Table 3:** The SUS questionnaire and results.

	Statements	Average Scores
Q1	I think that I would like to use this system frequently.	4.3
Q2	I found the system unnecessarily complex.	1.2
Q3	I thought the system was easy to use.	4.1
Q4	I think that I would need the support of a technical person to be able to use this system.	1.8
Q5	I found the various functions in this system were well integrated.	4.1
Q6	I thought there was too much inconsistency in this system.	2.3
Q7	I imagine that most people would learn to use this system very quickly.	4.1
Q8	I found the system very cumbersome to use.	2.2
Q9	I felt very confident using the system.	4.2
Q10	I needed to learn a lot of things before I could get going with this system.	1.9

## Data Availability

The original data presented in the study are openly available in the provided repository links.
